# Our Correspondence

**Published:** 1870-04

**Authors:** 


### Our Correspondence.

ELMIRA, N. Y. FEB. 14, 1870.

DR. UP DE GRAFF,

*Dear Sir*:—Inclosed I send you a quantity of circulars, certificates and agreements from Sayre & Co., of New York. You will at once see that they are on a par with every other swindling medicine concern of that great city Isn't their circular, addressed to me, affectionate? "Esteemed Friend!" Wonder when he formed my acquaintance?" Guess he won't think quite so much of me when he sees this. He is very kind to offer me four whole boxes of his consumption cure for ten dollars, with an agreement to return it if I'm not cured, don't you think so? How under the sun can he afford to make such an extravagant offer, I want to know? Please give him fits in the BISTOURY, for I know of several that have sent their money to this concern without having received any good whatever. Yours Truly,

T. N. L.

—Nothing is necessary to be said in connection with this letter. Any man who transacts business as do Sayre & Co., by sending agreements to refund money if a cure is not performed, and by mailing circulars and samples of medicines to the afflicted, are not to be trusted. No responsible person would dare to issue such agreements and circulars as were inclosed to T. N. L.

HORNELLSVILLE, Jan. 5, '70.

*Dear Dr*:—Please answer in the next number of your valuable Magazine, what kind of *Ether* is used for removing *tape worm*, also the dose.—Don't you think ⅔ of an ounce a heroic medication. I have a friend who is troubled with this worm, if the receipt is correct he is to try it. Affectionately Yours,

JAMES H——.

—Sulphuric Ether is meant. We have never tried the remedy. The dose must be correct else it would not be recommended by the *Gaz. Med. de Paris*. We can give you a formula that we have *never known to fail* in bringing away the tape worm. We will furnish it if you write us. It is not safe to make public as it might be abused.

WELLS, PA., Dec. 5th, 1869.

THAD. S. UP DE GRAFF, M. D.

I would like to solicit a little information from the columns of the BISTOURY. About three months since, I had a dark spot come before my left eye; it is about the bigness of a head of a pin, and about fourteen inches from my eye; it does not effect my sight, but keeps constantly before it. I am getting alarmed about it and would like to have you inform me what it is and what I had better do for it.

Truly Yours, L. S.

—You have some difficulty of the retina—hard to tell what, without seeing you. Better consult some capable surgeon and have the eye examined ophthalmoscopically.

---

CLEVELAND, O., Dec. 24 1869.

THAD. S. UP DE GRAFF, M. D., EDITOR BISTOURY, ELMIRA. N. Y.

*Dear Sir:*—Enclosed please find one dollar for BISTOURY. I don't know how you came to send it to me, but this I do know, since we began to *read* it, no periodical finds a more hearty welcome on our family reading table. It is full of kernels, the chaff having been winnowed out. Plain truths as you tell them must do good. Let the BISTOURY come.

Truly Yours, J. E. C.

—We publish this as a sample of the many letters we have received of this character. This one is selected because it is short and to the point.

---

CORNING, N. Y., Feb. 12, 1870.

DEAR BISTOURY:—I presume it is about time for you to be getting out "copy" for your next issue. I therefore scribble a few lines to you to say how delighted I am over a letter and your answer to the same, in the January number. A wife and mother, writing you from Syracuse, complains, or rather asks your advice with reference to a "remedy" for the abuse of the marriage relation.

Do you know, BISTOURY, I have often thought that *some* journal should have something to say upon this subject, and my heart leaped for joy when I saw that BISTOURY had the courage to open the subject and set people to thinking about it. There is more real suffering in the world from this abominable abuse than from all other causes combined. I firmly believe it, for I am not only a sufferer myself, but have listened to the complaints of many of my married friends. It is much more common than the public have any idea of. I could recite cases of sickness and terrible suffering, that have come under my observation that would terrify your readers, were it proper to place the history of them in print.

I write you this letter simply to enclose money for subscriptions for the following victims of their husband's lust, to thank you for the article written, to ask you to continue to write down the abuse and also to ask whether you will publish an article from my pen upon the subject. Very truly, your friend,

A WIFE.

—You write well—let us see the article and if suited to our columns it will be published. Wives are much more capable to write down this evil than we are. We will publish any well written and decent article upon the subject, and solicit our lady readers to furnish us with such.

Come, ladies, defend yourselves.

---

U. S. CONSULATE FOR THE
DUCHY OF BRUNSWICK.
JANUARY 23d, 1870.

MY DEAR DOCTOR:—* * * * * * The German diet, for a steady one, I find somewhat heavy, requiring a more powerful digestive apparatus than I am in possession of. But it seems to be a healthy one, at least so I infer from the seedy appearance of the desciples of Æsculapius here; for a more wretched class of creatures I never saw. I could not help contrasting them with the sleek and thriving gentlemen of the same profession on the other side of the water. * * * * *

The German tongue is very difficult to acquire, but I like it well, and am now able to carry on an ordinary conversation in that language.

Both numbers of the BISTOURY were received and have been carefully read. They are very interesting to me, and wish that they might be introduced into every family in America. I like the little periodical because of the simplicity of the language used in giving grand medical and physiological information—so simple as to be easily understood by the non-professional reader. * * * *

The Germans are a very peculiar people. As they were two hundred years ago, so are they now. When "Hannes" goes to mill he places a stone in one end of the sack and the grist in the other, simply because his great-great-grandfather did so before him. They are a conservative people and don't approve of inovations. A man here was never known to have any other trade, business or profession than that of his father; if Handenschancker sells beer, you know at once that his ancestor did also, in the time of Gambrinus. * * * The other day I saw a milk cart pass, drawn by a donkey, a dog and a woman—rather a mixed team eh? The woman did all the work of course, drawing cart, donkey and all. Now, you may rest assured that the cart or its father before it, was never drawn by any other sort of a team. * *

I send herewith a couple of papers, which I think you will enjoy reading.

Fraternally yours, D. W. C. S.

We clip the above few items from our friend's letter; they were not intended for publication, but we thought them of sufficient interest to our readers to warrant giving them publicity. We run the risk of a scolding from their author for the liberty taken with his letter.

### MEDICAL STAFF RANK.

Much is being written by medical men condemnatory of the present rank of surgeons in the government employ. Medical men are not sufficiently rewarded for their services. They are or should be, liberally educated men and gentlemen, who have charge of certainly, one of the most important branches of the service, yet are ranked and paid far below other officers of the Army. A Lieutenant, far the inferior of the assistant Surgeon, in point of education, culture and executive ability, with much less labor attached to his duties as an officer, rises rapidly to the rank of Captain, Major, Colonel, or even General; with pay and emoluments too, corresponding to his rank; while the surgeon attains the rank of major, and in rare instances, that of Lieut. Col., with the pay only, of a full surgeon, which is that of a Captain or Major of Infantry. This is wrong. Medical men should claim rank and pay equal to the field officers, and should be entitled to promotion as rapidly as their skill, ability or services may entitle them to such consideration. At present no inducement is offered our best medical talent to enter the army or navy. The pay is less than that of an ordinary practitioner of medicine in the country, while the rank does not equal that of the most inferior of our officers. Let physicians everywhere, use their influence through their medical societies, and by other means that may seem expedient, to have Congress establish a proper grade and pay for members of the medical branch of our service.

